# Microlearning to teach geriatric principles in hospitals: a systematic review and meta-analysis

**DOI:** 10.1093/ageing/afag129

**Published:** 2026-05-17

**Authors:** Carol L Hunter, Keerti Paida, Tim J Wilkinson, Kamal Masaki, Janani Thillainadesan

**Affiliations:** Department of Geriatric Medicine, Concord Repatriation General Hospital, Concord, New South Wales, Australia; Concord Clinical School, Sydney Medical School, Faculty of Medicine and Health, University of Sydney, Camperdown, New South Wales, Australia; Department of Geriatric Medicine, Concord Repatriation General Hospital, Concord, New South Wales, Australia; Department of Medicine, University of Otago, Christchurch, New Zealand; Department of Geriatric Medicine, John A. Burns School of Medicine, University of Hawai'i System, Honolulu, Hawaii, USA; Concord Clinical School, Sydney Medical School, Faculty of Medicine and Health, University of Sydney, Camperdown, New South Wales, Australia; Department of Geriatric Medicine and Centre for Education and Research on Ageing, Concord Repatriation General Hospital, Concord, New South Wales, Australia

**Keywords:** microlearning, microeducation, postgraduate healthcare professionals, geriatric medicine, older people, systematic review

## Abstract

**Background:**

Hospital-based education in geriatric medicine is often limited in availability and accessibility. Microlearning is defined as short, on-the-go, focused educational interventions (<15 minutes) and offers a practical way to deliver key geriatrics concepts in time-constrained environments. This systematic review investigated the effectiveness of microlearning to learn geriatric medicine principles for hospital-based clinicians.

**Methods:**

This systematic review with meta-analysis (PROSPERO: CRD42023422522) involved a comprehensive search across five databases. Studies evaluating microlearning interventions for hospital-based clinicians (medical, nursing and allied health) were included. Two independent reviewers conducted title/abstract screening and full-text review. Effectiveness was assessed using Kirkpatrick model, which evaluates educational outcomes across four levels: participant reaction, learning, behaviour and clinical practice. Data were synthesised narratively, and meta-analysis conducted using random-effects model. Study quality and risk of bias were assessed using Medical Education Research Quality Instrument and Newcastle–Ottawa scale-education.

**Results:**

Of 15 232 articles retrieved, 15 met inclusion criteria, mostly pre-post implementation studies (11/15, 73%). Common interventions included bedside teaching (6/15, 40.0%), pocket cards (5/15, 33.3%) and e-modules (4/15, 27%), focusing on delirium (9/15, 60%) and dementia (3/15, 20%). Of 40 educational outcomes measured, 90% showed positive results, and 30% were statistically significant. Meta-analysis indicated significant improvements in delirium knowledge (SMD 0.80, 95% CI 0.49–1.10, *P* < .00001) and recognition (SMD 0.91, 95% CI 0.10–1.72, *P* = .03).

**Conclusion:**

Microlearning shows promise as an effective educational intervention for learning geriatric medicine principles, particularly recognising delirium. Further research is needed to assess impact on patient outcomes and guide implementation in current training programs.

## Key Points

- Fifteen studies evaluated hospital-based microlearning for teaching geriatric principles to healthcare professionals.
- Microlearning interventions most commonly targeted delirium and dementia, improving knowledge, recognition and documentation.
- Meta-analysis showed that microlearning significantly improved both knowledge and recognition of delirium.

## Introduction

The ageing population and shortage of geriatricians has driven the need to upskill all healthcare professionals in geriatric competencies. While nearly all clinicians will encounter older adults in their practice, most older adults will never meet a geriatrician [1]. Shortages in geriatricians exist globally, highlighting the need for innovative and scalable educational approaches that equip the healthcare workforce to provide safe and effective care for older adults.

Microlearning has emerged as a promising educational intervention that could help bridge this gap [2]. First characterised by Theo Hug in 2005 [3], microlearning refers to short, focused learning sessions targeting one specific objective at a time, designed to be accessible anytime, anywhere. This method has proven effective in avoiding cognitive load [4]. While microlearning has gained traction in the age of rapid technological advancement and a shift away from traditional textbook- and classroom-based learning, it does not exclusively require technology as the medium by which it is delivered [3].

Increased access to electronic devices and software improvements have facilitated microlearning’s expansion across various industries, including health professions education, where its application remains relatively recent [5–7]. A scoping review of its potential role in health professions education identified just 17 relevant studies published between 2011 and 2019, all of which delivered microlearning interventions in under 15 minutes [5]. The range of learning activities that encompass microlearning remains broad and continually evolving. Recent examples include gamifying pharmacological concepts in pharmacy education [8], near-peer teaching in 10-minute intervals in surgical specialties [9] and online visual learning for skin lesion identification amongst nursing practitioners [10].

Traditional geriatric medicine education often involves lengthy, multicomponent programs that may not engage busy health professionals effectively [11]. Given their demanding schedules and varying levels of interest in geriatric medicine [12, 13], a more concise, targeted approach like microlearning may be better suited to their learning needs. Today’s health professional learners are characterised by their tech-savviness and preference for active, on-the-go learning, and may benefit more from microlearning’s concise, focused approach than from traditional methods [7, 14–16]. Given these challenges, this systematic review aims to explore the effectiveness of microlearning in geriatric medicine education for hospital-based healthcare professionals.

## Methods

This systematic review was prospectively registered with the International Prospective Register of Systematic Reviews (PROSPERO, registration number CRD42023422522) and follows the Preferred Reporting Items for Systematic Reviews and Meta-Analyses (PRISMA) guidelines for reporting [17].

### Data sources and search strategy

A comprehensive literature search was performed using the Population, Intervention, Comparator, Outcome (PICO) framework. The search strategy was optimised with the help of a university research librarian and tailored to each database. The search was designed to capture all relevant microlearning interventions from inception to April 2023, recognising the diversity of activities that fall under this broad definition. The full search strategy for MEDLINE is presented in Appendix 1. PubMed (MEDLINE), Embase, PsycINFO, CINAHL and Scopus were searched for English-language publications without date restrictions. The searches were conducted in May 2023 and updated in May 2024 prior to data analysis. Unpublished works (including theses and conference proceedings) were excluded. Reference lists of retrieved articles and relevant systematic reviews were searched.

### Eligibility criteria and study selection

Following removal of duplicate citations and title/abstract screening, subsequent full-text review was undertaken by two independent reviewers (C.H. and K.P.). The senior author (J.T.) was involved in resolving any disagreements.

Studies were included if: (i) the target population involved clinicians working in hospitals in medicine, nursing, pharmacy or allied health; (ii) they evaluated an educational intervention that included microlearning; (iii) they were undertaken in a hospital-based setting; and (iv) they reported one or more learning outcomes that could be classified by the Kirkpatrick model [18].

### Data extraction and analysis

Data were independently extracted by two reviewers (C.H. and K.P.) and input into a standard data entry form. Data extracted included study year, author(s), country, study design, study setting, target audience, sample size, learning topic, educator, educational intervention features including microlearning and control interventions, outcomes of interest, measuring tools and results. These data were reported narratively and summarised in tables. Learning outcomes were appraised using Kirkpatrick’s four-level model: (i) learner reaction/satisfaction, (ii) knowledge and skills acquisition, (iii) change in behaviour/practice and (iv) change in health outcomes [18]. Meta-analysis was conducted for outcomes reported in at least two studies. Given the diversity of settings and study design, we anticipated heterogeneity and thus used a random-effects model and Hedges’ g measure of effect size for the meta-analysis using SPSS Statistics Version 26.0. After synthesis of the findings, we sought feedback from our institution’s Patient and Stakeholder Advisory Board to inform the relevance and implications of our findings.

### Assessment of study quality

The methodological quality of the studies was assessed using the Medical Education Research Quality Instrument (MERSQI) (range score: 5–18) [19], and the risk of bias evaluated using the Newcastle–Ottawa scale-education (NOS-E) (range score: 0–6) [20]. These tools were selected as they have been validated as complementary tools for appraising methodological quality of medical education research and have high inter-rater reliability [21]. Two reviewers (C.H. and K.P.) independently undertook these assessments, and any disagreements were resolved through discussion with the senior author (J.T.). Where a study reported multiple types of learning outcome measures, all outcome types were scored, and the highest score was recorded. For example, if a study reported both self-reported outcomes (scoring 1 on the MERSQI scale) and objective outcomes such as multiple-choice test scores (scoring 3 on the MERSQI scale), the higher score of 3 was used for the MERSQI assessment. There are no defined cut-off values differentiating high- and low-quality studies; however, *a priori* studies have considered MERSQI scores of 14 or greater as high quality [22]. Given NOS-E scale ranges from 0–6, we defined low-quality studies as scoring 0–2, medium-quality scoring 3–4 and high-quality scoring 5–6.

## Results

### Search results

**Figure 1:**
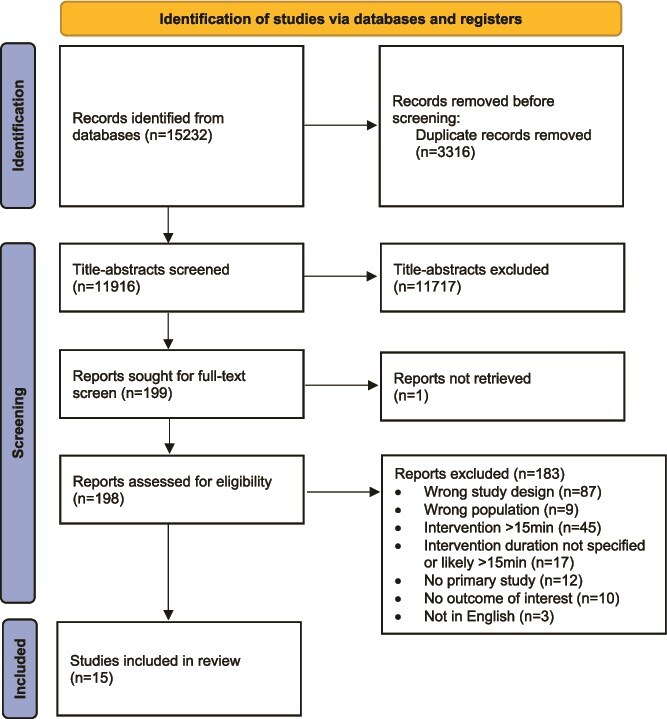
PRISMA flow diagram of study selection process for systematic review.

Out of 15 232 retrieved articles, 3316 duplicates were removed. The remaining 11 916 articles were screened for eligibility and 198 were included for full-text review. In total, 15 studies met the inclusion criteria for this review. The PRISMA flowchart of the study selection process, including reasons for exclusion, is shown in Figure 1.

### Study characteristics

The study characteristics are summarised in Table 1. The majority of studies were pre-post implementation studies (*n* = 11, 73.3%), with remaining study designs including retrospective case–control study (*n* = 1, 6.7%), single group post-implementation study (*n* = 2, 13.3%) and pre-post time series cluster randomised controlled trial (*n* = 1, 6.7%). Most studies were conducted in the USA (*n* = 5, 33.3%), followed by Canada and Australia (both *n* = 3, 20.0%).

**Table 1:** Characteristics of studies of microlearning interventions (*n* = 15).

Author, publication year, country	Study design	Study setting, target audience/learner participants (*n* = xx), sample size (SS)	Educational topic	Microlearning intervention	Other intervention components (non-microlearning)	Control intervention (if applicable)
Baluku et al. [23] 2023 Uganda	Pre-post implementation study	Setting: ICU/HDU Target: nurses SS: 29	Impact of an educational intervention on delirium knowledge and practice	- 2x 5-minute videos showing how CAM-ICU assessment tool is used - Case study scenario - Folder containing delirium pamphlets and pocket card, assessment forms, set of case study scenarios	- 1.5 hour face-to-face session offered twice per day for 3 weeks to capture all staff	No formal educational intervention specified in the study
Bush et al. [24] 2022 Canada	Pre-post implementation study	Setting: palliative care unit Target: nurses, physicians, pharmacists, allied health (*n* = 77) SS: 77	Delirium management in palliative care	- 15 minute face-to-face on antipsychotic monitoring in delirium (nurses only) - 3 x self-directed online e-modules 10–15 minutes each (one for nurses only, two for all staff) - Summary sheet in all patient binders - Guideline algorithm on a wall poster in palliative care unit rooms	- Face-to-face training providing core delirium education, orientate to guideline content, and e-Learning modules. For all healthcare team. Provided by members of the interprofessional guideline adaptation group (45 minutes). Followed by additional face-to-face session for nurses only immediately afterwards (15 minutes) - 1 x self-directed online e-module 30–40 minutes (for all staff)	No formal educational intervention specified in the study
Campbell et al. [25] 2011 USA	Retrospective case–control study	Setting: single-centre urban hospital Target: physicians SS: unclear how many physicians involved; total of 81 patient files reviewed	PEG tube placement in terminal-stage dementia	- ‘Just in time’ documentation in patient file by intervention team (dietitian, speech pathologist, palliative care) that recommends no PEG where appropriate	Nil	No formal educational intervention specified in the study
Choi et al. [26] 2020 USA	Pre-post implementation study	Setting: surgical-trauma unit Target: nurses SS: 23	Delirium recognition in surgical-trauma intermediate care setting	- 10-minute didactic presentation - 8-minute video-recorded simulation - 15-minute bedside coaching	Nil	No formal educational intervention specified in the study
Detroyer et al. [27] 2016 Belgium	Pre-post implementation study	Setting: 20 adult inpatient units of a university hospital, wards included medical/surgical/geronto-psychiatric/rehabilitation Target: all healthcare providers SS: 55 nurses, 2 physiotherapists, 2 occupational therapists	Effect of delirium e-learning tool on delirium recognition, knowledge and strain in caring	- Self-learning delirium e-modules, 11 in total lasting 5–15 minutes each, available to staff for 2 months, covering detection, prevention and management	- Live information session on how to use e-learning tool (1 hour duration)	No formal educational intervention specified in the study
Detroyer et al. [28] 2018 Belgium	Pre-post implementation study	Setting: geriatric inpatient units of a university hospital. For patient outcomes: Dutch >70 years old, without severe hearing/visual disability, MRO isolation, unable to hold conversation, very poor health. Target: nursing staff SS: 17 nurses, total of 160 patient outcomes assessed (81 control, 79 intervention)	Effect of delirium e-learning tool on patient and nursing outcomes in geriatric unit	- Self-learning delirium e-modules, 11 in total lasting 5–15 minutes each, available to staff for 3 months, first 6 modules compulsory to complete, covering detection, prevention and management	- Live information session on how to use e-learning tool (1 hour duration)	No formal educational intervention specified in the study
Gesin et al. [29] 2012 USA	Pre-post implementation study (3 phases)	Setting: surgical trauma ICU (STICU) with 29 beds in community teaching hospital Target: nurses with at least 1 year of experience in STICU SS: 19	Effect of multifaceted educational tool on delirium recognition and knowledge in surgical-trauma ICU	- Phase 2: Provision of Intensive Care Delirium Screening Checklist (ICDSC) (this is an 8-item 1-page assessment tool) and validating article (estimated reading time <15 minutes)	- Phase 3: 30-slide live presentation by pharmacist and bedside demonstration of ICDSC by most experienced nurse	Phase 1: no formal educational intervention specified in the study
Hobday et al. [30] 2017 USA	Pre-post implementation study	Setting: acute hospital wards in rural and metropolitan mid-Western states USA Target: nursing assistants (NA) and allied health workers (AHW) SS: 25 NA/AHW across 10 hospitals in 5 states (6 rural, 4 metropolitan)	Feasibility and utility of online dementia training tool for nursing aides and allied health workers in acute wards	- Self-learning e-modules [CARES Dementia-Friendly Hospital program (CDFH)], 4 in total lasting 5–15 minutes each	Nil	No formal educational intervention specified in the study
Hung et al. [31] 2023 Canada	Single group post-implementation study	Setting: 10 hospitals and 10 long-term care homes Target: nurses, care aides, physicians, rehabilitation staff SS: 3025 (65% hospital-based, 35% community-based), 40% followed through to post-intervention phase	Impact of an online dementia education game on dementia knowledge	- Dementia education game available on mobile phones/tablets/computers, separated into short games lasting ~2 minutes each	Nil	N/A
Ilievski et al. [32] 2023 Australia	Pre-post implementation study	Setting: 300-bed regional hospital in Australia Target: staff who attended at least 1 Clinical Aggression Response Team (C-ART) call SS: unclear how many staff invited to participate; data from 57 C-ART calls for 23 patients included pre-intervention, 32 C-ART calls for 14 patients post-intervention	Impact of a multimodal intervention on management of behavioural emergencies in cognitive impairment	- Pre-reading material sent to doctors at start of rotation emphasising 2 major points on diagnostics of behavioural disturbance and providing appropriate handover - Ward and case-based brief education sessions for nursing staff targeting 2 key concepts of appropriate handover and initiating de-escalation strategies	- 1 hour training session for doctors - Updated sedation guidelines developed and circulated	No formal educational intervention specified in the study
McCrow et al. [33] 2014 Australia	Pre-post time series cluster randomised controlled trial	Setting: 4 high-risk delirium areas (critical care, orthopaedics, medical, surgical) in 3 hospitals (250–300 beds each) in South-East Queensland Target: registered nurses (part-time or full-time) in 4 high-risk delirium areas (critical care, orthopaedics, medical, surgical) in 3 hospitals SS: 147 (segregated into 12 clusters—i.e. 4 wards at 3 hospitals); 6 clusters received intervention (*n* = 75) and 6 received control (*n* = 72)	Effect of educational website on delirium knowledge and recognition in acute care nurses	- Website including video vignettes, self-assessment questions, downloadable flow charts, links to external sites, available for access for 5 weeks. Duration of individual tasks not specified; however, website was ‘not developed in a modular form as many online courses are structured’. Was instead developed using ‘cognitive constructivist principles to allow an individualised approach to website navigation’.	Nil	No formal educational intervention specified in the study
McMillan et al. [34] 2023 UK	Pre-post implementation study (3 phases)	Setting: medical ward at single hospital site Target: medical prescribers SS: unclear how many prescribers involved; 157–167 patient files reviewed per phase of study	Impact of an educational intervention on PRN analgesia and laxative use	- Poster displayed and electronically circulated (phase 1)—placed on each medical ward to serve as a visual reminder - Teaching presentation which is also electronically circulated (phase 2). Duration not specified.	Nil	No formal educational intervention specified in the study (phase 0)
Thillainadesan et al. [6] 2023 Australia	Pre-post implementation study	Setting: vascular surgery unit of tertiary teaching hospital Target: resident medical officers SS: 8 pre-intervention and 12 post-intervention; 302 patient charts reviewed (150 pre-intervention, 152 post-intervention)	Effect of microlearning on perioperative comprehensive geriatric assessments	- Introduction of geriatrician within vascular surgery team providing 15-minute ward-based microlearning on topics of: (1) screening for delirium and cognitive impairment using 4AT and (2) screening for frailty using CFS - Provision of lanyard cards and mobile app resources	- Ward-based experiential methods - Other CGA components such as deprescribing and advance care planning taught on-the-go during ward rounds	No formal educational intervention specified in the study
Yan et al. [35] 2019 USA	Pre-post implementation study	Setting: 15-bed ICU servicing 180-bed hospital with mix of rural/urban patients across medical, surgical, cardiac, neurological domains Target: registered nurses (RN), nurse practitioners (NP), intensivists SS: 44 RNs, 2NPs, 1 intensivist (of a total pool of 55, 12 and 7, respectively)	Effect of a multimodal educational module on adherence to pain, agitation and delirium protocols in intensive care units	- Research articles provided to staff - Medical- and nursing-specific posters in relevant patient care areas - Point-of-care reminders on each computer monitor - Step-by-step checklists at each computer station	- Online education module to be completed within 4 weeks of unspecified length	No formal educational intervention specified in the study
Zhang et al. [36] 2015 Canada	Single group post-implementation study	Setting: application available via online hosting portal and Android Play store Target: psychiatry residents, allied health staff at university hospital SS: 10 psychiatry residents, 9 allied health staff	Development and feasibility of a delirium educational application for psychiatry doctors and allied health staff	- Smartphone app with overview of definitions, causes, assessment tools, interventions, patient/family information, links to other website resources	Nil	N/A

Abbreviations: C-ART, Clinical Aggression Response Team; CAM-ICU, Confusion Assessment Method – Intensive Care Unit; CDFH, CARES Dementia-Friendly Hospital Program; CFS, Clinical Frailty Scale; CGA, Comprehensive Geriatric Assessment; ICDSC, Intensive Care Delirium Screening Checklist; ICU/HDU, Intensive Care Unit/High Dependency Unit; MRO, Multi-Resistant Organism; NA/AHW, Nursing Assistant/Allied Health Worker; NP, Nurse Practitioner; PEG, Percutaneous Endoscopic Gastrostomy; RN, Registered Nurse; STICU, Surgical Trauma Intensive Care Unit; 4AT, 4 ‘A’s Test

Studies were performed in adult clinical wards (*n* = 7, 46.7%), intensive care units (*n* = 4, 26.7%), surgical/trauma units (*n* = 4, 26.7%), geriatrics ward (*n* = 1, 6.7%), palliative care unit (*n* = 1, 6.7%) and psychiatry unit (*n* = 1, 6.7%). Target populations for delivery of microlearning interventions included nursing staff (*n* = 11, 73.3%), physicians (*n* = 8, 53.3%), allied health workers (*n* = 4, 26.7%) and pharmacists (*n* = 1, 6.7%).

### Educational interventions

The microlearning intervention formed the sole intervention in seven of the included studies (46.7%). In the remaining eight studies (53.3%), the microlearning intervention was included as a component of a multicomponent educational intervention. In all studies where a control group was present (*n* = 13, 86.7%), the control group did not report on what educational intervention existed prior to the microlearning intervention and received no additional or alternative educational intervention during the study period.

Microlearning interventions included direct/bedside teaching (*n* = 6, 40.0%), pamphlet sheets/pocket cards (*n* = 5, 33.3%), online e-modules (*n* = 4, 26.7%), pre-reading material (*n* = 3, 20.0%), wall posters (*n* = 3, 20.0%), video recordings (*n* = 2, 13.3%), case study scenarios (*n* = 2, 13.3%), mobile game/applications (*n* = 2, 13.3%), computer-based alerts (*n* = 2, 13.3%) and website (*n* = 1, 6.7%).

The other educational interventions combined with microlearning included direct/bedside teaching (*n* = 8, 53.3%), online e-modules (*n* = 2, 13.3%) and extended reading materials (*n* = 1, 6.7%), all of which lasted longer than 15 minutes or contained learning targeted at more than one objective.

The learning topics addressed by these educational interventions were delirium (*n* = 9, 60.0%), dementia (*n* = 3, 20.0%), behavioural management (*n* = 1, 6.7%), analgesia and laxative use (*n* = 1, 6.7%) and perioperative comprehensive geriatric assessments (*n* = 1, 6.7%). Of these learning topics, 8 were taught via self-directed resources (53.3%), 2 were delivered via an educator (13.3%) and 5 were a combination of both modalities (33.3%).

### Kirkpatrick outcome evaluation

The reported outcomes and findings are shown in Table 2. Across the 15 studies, there were 40 outcomes of interest measured. None of the included studies directly compared microlearning with traditional didactic teaching methods. In all studies with a control group, the control condition involved no formal educational intervention.

**Table 2:** Outcomes of interest, measurement tools, results and effectiveness (as per Kirkpatrick model) of microlearning interventions.

Author, publication year, country	Topic	Outcomes	Outcome measurement tool	Outcome results	Kirkpatrick level
Baluku et al. [23] 2023 Uganda	Impact of an educational intervention on delirium knowledge and practice	- Delirium knowledge	- Multiple-choice and Likert questions developed by investigators	- Pre-intervention: mean score 10.7, SD 12.36 - Post-intervention: mean score 19, SD 0.94 *P*-value <.001	2
		- Nursing practice behaviours related to delirium detection	- 6x yes/no nurse-reported questions regarding awareness and behaviours relating to department-specific delirium practices	- Pre-intervention: mean score 2, SD 0.83 - Post-intervention: mean score 4, SD 0.35 *P*-value <.001	2
Bush et al. [24] 2022 Canada	Delirium management in palliative care	- Guideline module completion >/=85%	- Retrospective pre-post chart audit	Overall online module completion rate 80.4% (*n* = 62/77) - Delirium 73% (*n* = 56/77) - Non-pharmacological 90% (*n* = 69/77) - Communication 88.5% (*n* = 68/77) - Pharmacological 70% (*n* = 54/77)	1
		- Documentation of delirium behaviours		- Pre: *n* = 16/20 (80%) - Post: *n* = 17/20 (85%)	3
		- Documentation of non-pharmacological interventions		- Pre: *n* = 0/20 (0%) - Post: *n* = 4/20 (20%)	3
		- Antipsychotic and benzodiazepine medication administration - Reduction in use of antipsychotics in delirium		Antipsychotic use - Intervention group: - 60% reduced regular use - Similar PRN use - 50% increased PRN midazolam	3
		- Team perceptions on accessibility, practicability, acceptability	- Pre-post implementation survey - Focus group or one-on-one interviews	- Accessibility 17/25 (68%) - Practicability 25/25 (100%) - Acceptability 17/25 (68%)	1
Campbell et al. [25] 2011 USA	PEG tube placement in terminal-stage dementia	Rates of inpatient PEG tube insertion pre- and post-educational intervention	- Chart audit	Control: 4/71 (5.6%) Intervention: 0/10 (10%) *P*-value .44	4
Choi et al. [26] 2020 USA	Delirium recognition in surgical-trauma intermediate care setting	- Delirium recognition knowledge by nursing staff	- Nurses’ Delirium Knowledge Questionnaire (NDKQ), score out of 25	- Pre: mean 18.65/25 (74.6%), SD 3.34 - Post: mean 22.87/25 (91.5%), SD 2.42 *P*-value <.001	2
		- Agreement in delirium recognition between junior and senior nursing staff	- Agreement measured by Nursing Delirium Screening Scale (Nu-DESC) as marked by evaluator nurse	- Pre: *n* = 5/23 (21.7%) - Post: *n* = 23/23 (100%)	2
		- Confidence in performing delirium assessment	- Confidence Scale (C-Scale) - 5 statements with 5-point Likert scale	- Pre: mean 16.43, SD 4.37 - Post: mean 22.91, SD 2.70 *P*-value <.001	1
Detroyer et al. [27] 2016 Belgium	Effect of delirium e-learning tool on delirium recognition, knowledge and strain in caring	- Completion of e-learning tool		Participation/completion: - *n* = 59 - Completed 0–6 modules: *n* = 19 (32.2%) - Completed 7–11 modules: *n* = 40 (67.8%); *n* = 26 (44.1%) completed ALL modules	1
		- Delirium recognition	- Case vignettes x4 (for delirium recognition [DR]) - score range 0 to 4	- Pre: mean 2.7/4 (67.5%), SD 1.1 - Post: mean 3.1 (77.5%), SD 0.9 *P*-value .04 No difference between high and low completion rate groups	2
		- Delirium knowledge	- Delirium Knowledge Questionnaire (DKQ)—developed by investigators, validated by independent expert panel, score range 0 to 35	- Pre: mean 28.3/35 (80.9%), SD 4.5 - Post: mean 31.7/35 (90.6%), SD 2.6 *P*-value <.001 Significant increased level within high completion rate group, *P* = .02	2
		- Strain in caring for delirium patients	- Strain of Care for Delirium Index (SCDI)—contains 20 items, score range 20 to 80 (higher score = greater difficulty managing delirium)	- Pre: mean 50.9/80 (63.6%), SD 9.2 - Post: mean 51.2/80 (64%), SD 8.4 *P*-value .46 No difference between high and low completion rate groups	2
Detroyer et al. [28] 2018 Belgium	Effect of delirium e-learning tool on patient and nursing outcomes in geriatric unit	- Completion of e-learning tool	–	- *n* = 17 - *n* = 15/17 (88.2%) completed 6 compulsory modules within 3 month implementation period - *n* = 2/17 (11.8%) completed 6 compulsory modules 1 month after implementation period - *n*= 3/17 (17.6%) completed >6 modules (i.e. also undertook optional modules)	1
		- Patient delirium prevalence, duration, severity, mortality (in-hospital and 12 months)	- Confusion Assessment Method (CAM)—for prevalence and duration (combination of face-to-face data collection and chart review) - Delirium Index (DI)—for severity, score range 0 to 21 (combination of face-to-face data collection and chart review) - Chart audit—for mortality	Prevalence: - Intervention: *n* = 17/79 (21.5%) - Control: *n* = 21/81 (25.9%) *P*-value .51 Duration: - Intervention: mean 4.2 days, SD 4.8 - Control: mean 4.9 days, SD 4.8 *P*-value .38 Severity: Linear mixed model analysis noted trend towards lower severity score in intervention vs. control cohort Difference estimate −1.59, 95% CI −3.37 to 0.19, *P* = .08 Mortality: - In-hospital for control vs. intervention - OR 0.85, 95% CI 0.20–3.66, *P* = .80 - 12-month for control vs. intervention—OR 0.75, 95% CI 0.33–1.71, *P* = .50	4
		- Nurses’ delirium recognition	- Case vignettes x4 with multiple choice answers [for delirium recognition (DR)]—score range 0 to 4	- Control: mean 3.1/4 (77.5%), SD 0.83 - Intervention: mean 3.1/4 (77.5%), SD 0.75 *P*-value 1.0	2
		- Nurses’ delirium knowledge	- Delirium Knowledge Questionnaire (DKQ)—developed by investigators, validated by independent expert panel, score range 0 to 35	- Control: mean 29.3/35 (83.7%), SD 2.6 - Intervention: mean 29.9/35 (85.4%), SD 3.2 *P*-value .43	2
Gesin et al. [29] 2012 USA	Effect of multifaceted educational tool on delirium recognition and knowledge in surgical-trauma ICU	- Delirium knowledge	- 10 multiple-choice questions delivered between each study phase (from a pool of 30 questions developed by investigators)	- Phase 1: mean 6.1/10 (61%), SD 1.4 - Phase 2: mean 6.5/10 (65%), SD 1.4 (*P* = .08 compared to P1) - Phase 3: mean 8.2/10 (82%), SD 1.4 (*P* = .001 compared to P1)	2
		- Accuracy of delirium recognition	- Agreement between ICDSC scores of validated investigator and participant	- Phase 1: K = 0.40, 95% CI 0.11–0.69 - fair, *n* = 22/32 (69%) - Phase 2: K = 0.62, 95% CI 0.39–0.69 - substantial, *n* = 26/32 (81%) - Phase 3: K = 0.74, 95% CI 0.69–0.95 - substantial, *n* = 23/26 (88%)	2
		- Perceptions on delirium assessment	- Participant-reported survey for perceptions	- Consider delirium to be challenging to assess in ICU Phase 1: *n* = 17/19 (89.5%), Phase 2: *n* = 15/19 (78.9%), Phase 3: *n* = 12/19 (63.2%), mean difference P1 to P3 –0.57 (*P* = .07) - Agree that ICDSC makes delirium easier to identify Phase 1: *n* = 11/19 (57.9%), Phase 2: *n* = 15/19 (78.9%), Phase 3: *n* = 17/19 (89.5%), mean difference P1 to P3 0.58 (*P* = .06)	1
Hobday et al. [30] 2017 USA	Feasibility and utility of online dementia training tool for nursing aides and allied health workers in acute wards	- Dementia knowledge	- 19-item multiple-choice and true/false knowledge test	- Pre: mean score *n* = 15.6/19 (82.2% +/− 10.71%) - Post: mean score *n* = 17.4/19 (91.6% +/− 6.08%) *P*-value <.001 80% demonstrated gain in knowledge (*n* = 20), 8% no change (*n* = 2), 12% decrease (*n* = 3)	2
		- Satisfaction with e-module program (CDFH)	- 9-item Likert scale questionnaire and 3 open-ended questions	- 100% (*n* = 25) agreed or strongly agreed that CDFH led to improved satisfaction including confidence in dementia care skills, gaining new ideas on how to care for those with dementia, improved understanding of cognitive changes in dementia - 96% (*n* = 24) agreed or strongly agreed that modules provided ‘right amount of information’	1
Hung et al. [31] 2023 Canada	Impact of an online dementia education game on dementia knowledge	- Delirium knowledge	- 10-item knowledge test	- Post-intervention: 90% scored 100% (*n* not provided)	2
		- Participant experience feedback	- 3-item learner experience test	- Games were fun: 90% agree (*n* not provided) - Games helped teach practical skills: 93% agree (*n* not provided) - Would recommend to others: 95% (*n* not provided)	1
Ilievski et al. [32] 2023 Australia	Impact of a multimodal intervention on management of behavioural emergencies in cognitive impairment	- Adherence to local C-ART guidelines	- Chart audit for documentation of frequency of C-ART calls	Multiple C-ART calls per patient: - Pre: *n* = 10 (43.5%) vs. Post: *n* = 7 (50.0%), OR 1.3 (0.34–4.93) *P*-value .70	4
		- Adherence to local C-ART guidelines	- Chart audit for documentation of aetiology of behaviours, use of communication tools, use of sedating psychotropics, physical restraints, de-escalation techniques	Aetiology documented: - Pre: *n* = 21 (36.8%) vs. Post: *n* = 21 (65.6%), OR 1.24 (0.4–4.2) *P*-value .73 Communication tools: - Pre: *n* = 34 (59.7%) vs. Post: *n* = 25 (78.1%), OR 1.7 (0.5–5.8) *P*-value .40 Psychotropics used: - Pre: *n* = 50 (87.7%) vs. Post: *n* = 27 (84.4%), OR 0.7 (0.2–2.4) *P*-value .62 Physical restraints used: - Pre: *n* = 22 (38.6%) vs. Post: *n* = 17 (53.1%), OR 1.8 (0.4–4.1) *P*-value .69 De-escalation techniques used: - Pre: *n* = 44 (77.2%) vs. Post: *n* = 24 (75.0%), OR 1.0 (0.3–3.6) *P*-value .98	3
McCrow et al. [33] 2014 Australia	Effect of educational website on delirium knowledge and recognition in acute care nurses	- Delirium knowledge (data collected pre-intervention [T1], immediately after intervention [T2] and 6–8 weeks after intervention [T3])	- Delirium Knowledge Questionnaire (DKQ)—developed by investigators based on Hare study, score range 0 to 34	Intervention: - T1: mean 19.6/34 (57.6%), estimate 19.67, 95% CI 18.74–20.58, t = 44.71, *P* < .001 - T2: 25.43/34 (74.8%), 3.95, 2.61–5.29, t = 5.83, *P* < .001 - T3: 25.62/34 (75.4%), 2.98, 1.42–4.54, t = 3.78, *P* < .001 Control: - T1: mean 20.05/34 (59.0%), estimate 20.05, 95% CI 19.15–20.95, t = 44.71, *P* < .001 - T2: 21.86/34 (64.3%), 1.81, 0.92–2.71, t = 4.08, *P* < .001 - T3: 23.02/34 (67.7%), 2.96, 1.86–4.07, t = 3.78, *P* < .001	2
		- Delirium recognition (data collected pre-intervention [T1], immediately after intervention [T2] and 6–8 weeks after intervention [T3])	- Delirium Recognition—2 sets of standardised vignettes with 5 case scenarios in each	Intervention: - T1: mean 2.93/5 (58.6%), estimate 2.92, 95% CI 2.64–3.21, t = 20.56, *P* < .001 - T2: 4.00/5 (80%), 0.64, 0.15–1.13, t = 2.56, *P* = .01 -T3: 3.26/5 (65.2%), 0.51, −0.05–1.08, t = 1.80, *P* = .07 Control: - T1: mean 2.85/5 (57%), estimate 2.85, 95% CI 2.54–3.16, t = 18.63, *P* < .001 - T2: 3.28/5 (65.6%), 0.43, 0.08–0.79, t = 2.44, *P* = .02 - T3: 2.67 (53.4%), −0.18, −0.57–0.22, t = −0.88, *P* = .38	2
		- Satisfaction with website resource	- Evaluation of Online Learning: Self-Assessment Questionnaire—27 items scored on 5-point Likert Scale (content, visual, technical aspects)	Satisfaction: - *n* = 49/61 (80%)—agreed website visually appealing - *n* = 51/61 (84%)—liked navigational features - *n* = 50/61 (82%)—found website flexible, relevant to clinical practice and interesting	1
McMillan et al. [34] 2023 UK	Impact of an educational intervention on PRN analgesia and laxative use	- PRN analgesia use which met WHO analgesic ladder criteria	- Prospective chart reviews performed with interventions introduced at 2 separate times points (phases 1 and 2)	PRN analgesia use meeting WHO criteria: - Pre-intervention (P0): *n* = 62/167 (37.1%) - P1: *n* = 92/159 (57.9%) - P2: *n* = 107/157 (68.2%) *P*-value <.005	3
		- Concurrent prescribing of laxatives for those on either opioid analgesia or those >65 years old		Laxatives not prescribed where indicated: - P0: *n* = 32/167 (19.2%) - P1: *n* = 30/159 (18.9%) - P2: *n* = 22/157 (14.0%)	3
Thillainadesan et al. [6] 2023 Australia	Effect of microlearning on perioperative comprehensive geriatric assessments	- Occurrence of 12 care processes for which resident medical officers are responsible	- Chart audit on documentation and screening practices	Documentation (pre vs. post): - Pain status: 92/150 (61.3%) vs. 105/152 (69.1%), *P* = .158 - Medications: 80/150 (53.3%) vs. 113/152 (74.3%), *P* < .001 - Alcohol/smoking status: 61/150 (40.7%) vs. 105/152 (69.1%), *P* < .001 - Functional status: 51/150 (34.0%) vs. 115/152 (75.7%), *P* < .001 - Mobility: 40/150 (26.7%) vs. 119/152 (78.3%), *P* < .001 - Cause for delirium where relevant: 8/10 (80%) vs. 12/14 (85.7%), *P* = .711 - Medical review post-fall where relevant: 3/6 (50.0%) vs. 4/7 (57.1%), *P* = .797 Screening (pre vs. post): - CFS: 0/150 (0.0%) vs. 98/152 (64.5%), *P* < .001 - Cognitive impairment: 12/150 (8.0%) vs. 116/12 (76.3%), *P* < .001 - Delirium: 3/150 (2.0%) vs. 105/152 (69.1%), *P* < .001 - Cognition in preadmission clinic: 7/47 (14.9%) vs. 17/30 (56.7%), *P* < .001 - Delirium in preadmission clinic: 10/47 (21.3%) vs. 15/30 (50.0%), *P* = .009	3
Yan et al. [35] 2019 USA	Effect of a multimodal educational module on adherence to pain, agitation and delirium protocols in intensive care units	- Knowledge of delirium protocol	- Knowledge: multiple-choice questions developed by investigators (5 in pre-intervention, 8 in post-intervention)	- Pre: mean *n* = 3.25/5 (65%, SD 18%) - Post: mean *n* = 6.56/8 (82%, SD 14%)	2
		- Compliance towards delirium protocol	- Compliance: retrospective and prospective chart reviews conducted 3 months before and after intervention implementation	- Pre: *n* = 25/34 patient charts reviewed (73.5%) - Post: *n* = 24/58 (41.4%) *P*-value <.01	3
Zhang et al. [36] 2015 Canada	Development and feasibility of a delirium educational application for psychiatry doctors and allied health staff	- Confidence utilising application	- 5-point Likert scale questionnaire	- Pre-application (but data collected retrospectively): *n* = 8/18 (44.4%) - Post-application: *n* = 15/18 (83.3%)	1

Abbreviations: C-ART, Clinical Aggression Response Team; C-Scale, Confidence Scale; CAM, Confusion Assessment Method; CDFH, CARES Dementia-Friendly Hospital Program; CFS: Clinical Frailty Scale; DI, Delirium Index; DR, Delirium Recognition; ICDSC, Intensive Care Delirium Screening Checklist; ICU, Intensive Care Unit; [N]DKQ, [Nurses’] Delirium Knowledge Questionnaire; Nu-DESC, Nursing Delirium Screening Scale; PEG, Percutaneous Endoscopic Gastrostomy; PRN, Pro Re Nata (as needed); SCDI, Strain of Care for Delirium Index; WHO, World Health Organisation

There was a total of 10 (25.0%) Level 1 outcomes assessed. These included module completion rates (*n* = 3/40, 7.5%), perception on acceptability/satisfaction of interventions (*n* = 5, 12.5%) and confidence in utilising tools/performing assessments (*n* = 2, 5.0%). These were assessed using pre-/post-intervention surveys (*n* = 6), completion rate auditing (*n* = 3) and confidence scales (*n* = 1). Of these outcomes, significant benefits were reported in one outcome (10.0%), namely clinician confidence in performing delirium assessment, measured using a five-question Likert scale [26]. The remaining nine outcomes (90.0%) reported positive findings, but statistical significance was either not demonstrated or not tested [24, 27–31, 33, 36].

Level 2 outcomes constituted 16/40 (40.0%) of total outcomes assessed. These included delirium/dementia knowledge (*n* = 7, 17.5%), delirium/dementia recognition (*n* = 5, 12.5%), delirium recognition accuracy (*n* = 2, 5.0%), strain in caring (*n* = 1, 2.5%) and delirium protocol knowledge (*n* = 1, 2.5%). These were assessed using knowledge questionnaires (*n* = 9), assessment scales (*n* = 3), case vignettes (*n* = 3) and self-reported questions/answers (*n* = 1). Of these outcomes, significant benefits were reported in 9 of 16 outcomes (56.3%). These outcomes all related to either delirium knowledge as measured with multiple-choice questionnaires [23, 26, 27, 29, 30, 33] or delirium recognition [33]. Of the remaining seven outcomes, five reported positive findings but statistical significance was either not demonstrated or not tested (31.3%) [26–28, 31, 35], while two reported no change in outcomes with respect to strain in caring for patients with delirium [27] and nursing ability to recognise delirium within a geriatrics unit [33].

Level 3 outcomes constituted 8/40 (20.0%) of total outcomes assessed. These included documentation of delirium rates, implementation of non-pharmacological behavioural management strategies, antipsychotic use, analgesia use, laxative use, perioperative comprehensive geriatrics assessment (CGA) and management, compliance to a delirium protocol and compliance to a clinical aggression response team (C-ART) protocol. All outcomes (100%) were assessed using prospective and/or retrospective chart audit. Of these outcomes, significant benefits were reported in 2 of 8 outcomes (25.0%). These outcomes were rates of appropriate analgesia use in older adults [34] and documentation/screening of perioperative CGA parameters [6]. A negative finding was reported in 1 outcome (12.5%), with a significant reduction in compliance to a delirium protocol in the intensive care setting after a microlearning intervention [35]. Of the remaining 5 outcomes, 4 (50.0%) report positive findings but statistical significance was not tested [24, 34], and 1 (12.5%) reported mixed adherence to a C-ART protocol in different aspects of auditing [32].

Level 4 outcomes constituted 6/40 (15%) of total outcomes assessed. These included patient delirium outcomes (prevalence, duration, severity, mortality) (*n* = 4, 10.0%), rates of percutaneous endoscopic gastrostomy (PEG) tube insertion (*n* = 1, 2.5%) and rates of patient C-ART calls (*n* = 1, 2.5%). All outcomes (*n* = 6, 100.0%) reported positive findings but statistical significance was not demonstrated [25, 28, 32].

### Meta-analysis

**Figure 2:**
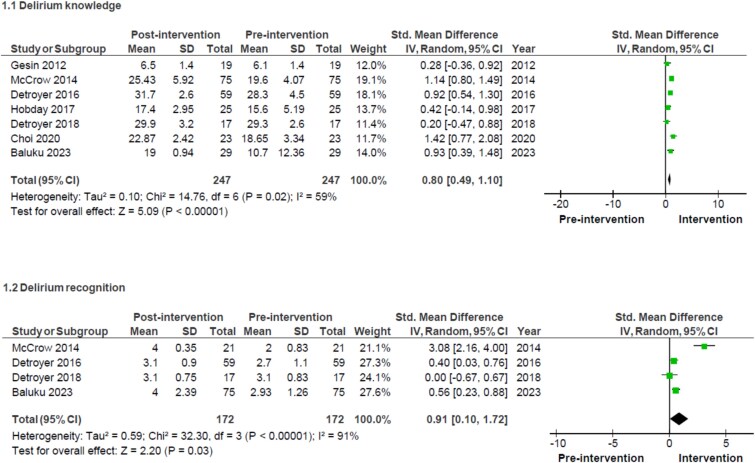
Meta-analysis of (1) delirium knowledge and (2) delirium recognition, measured using standard mean difference and random effects model, showing 95% confidence intervals.

Meta-analysis was conducted on outcomes of delirium knowledge [23, 26–30, 33] and delirium recognition [23, 27, 28, 33]. This showed statistically significant increases in both delirium knowledge (SMD 0.80, 95% CI 0.49–1.10, *P* < .00001) and delirium recognition (SMD 0.91, 95% CI 0.10–1.72, *P* = .03) in the post-intervention cohorts (Figure 2). In each of the studies included for meta-analysis, the control arm was receiving no formal educational intervention.

### Assessment of study quality

The overall methodological quality of the studies was moderate (Table 3). The average MERSQI score was 11.17, with scores ranging from 5.5 to 14.5 (median 11.5). The average NOS-E score was 2.13, with scores ranging from 0 to 6 (median 2.0). There was complete inter-rater agreement with both tools used.

**Table 3:** Assessment of study quality as determined using MERSQI and NOS-E.

	Study design	Sampling—institutions	Sampling—response rate	Data type	Validity	Data analysis—sophistication	Data analysis—appropriateness	Outcome	Representativeness	Comparison group—selection	Comparison group—comparability	Study retention	Blinding	Risk of bias
Baluku et al. [23]	▲△△	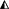 △△	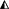 △△	▲▲▲	▲△△	▲△△	▲△△	▲▲▲	▲	△△△	△△△	△	△	Unclear
Bush et al. [24]	▲▲△	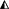 △△	–	▲▲▲	–	▲▲△	▲△△	▲▲▲	▲	△△△	△△△	△	△	Unclear
Campbell et al. [25]	▲ 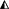 △	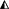 △△	▲ 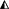 △	▲▲▲	▲△△	▲▲△	▲△△	▲ 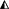 △	▲	△△△	△△△	▲	▲	Unclear
Choi et al. [26]	▲ 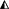 △	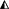 △△	▲ 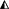 △	▲▲▲	▲△△	▲▲△	▲△△	▲▲△	▲	△△△	△△△	▲	▲	Unclear
Detroyer et al. [27]	▲ 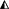 △	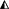 △△	▲ 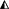 △	▲▲▲	▲△△	▲▲△	▲△△	▲▲▲	▲	△△△	△△△	▲	▲	Unclear
Detroyer et al. [28]	▲ 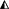 △	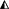 △△	▲ 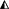 △	▲▲▲	▲△△	▲▲△	▲△△	▲ 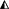 △	▲	△△△	△△△	▲	▲	Unclear
Gesin et al. [29]	▲ 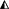 △	▲ 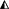 △	▲ 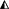 △	▲▲▲	▲△△	▲▲△	▲△△	▲ 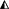 △	△	△△△	△△△	▲	▲	Unclear
Hobday et al. [30]	▲▲▲	▲ 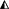 △	▲ 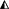 △	▲▲▲	▲△△	▲▲△	▲△△	▲ 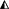 △	▲	▲△△	▲▲△	▲	▲	Unclear
Hung et al. [31]	▲ 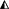 △	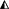 △△	▲ 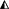 △	▲▲▲	▲△△	▲▲△	▲△△	▲▲△	△	△△△	△△△	▲	▲	Low
Ilievski et al. [32]	▲△△	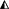 △△	–	▲△△	–	▲△△	▲△△	▲△△	△	△△△	△△△	△	△	Unclear
McCrow et al. [33]	▲ 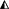 △	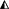 △△	–	▲▲▲	–	▲▲△	▲△△	▲▲△	△	△△△	△△△	△	▲	High
McMillan et al. [34]	▲ 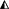 △	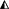 △△	▲ 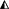 △	▲△△	▲△△	▲▲△	▲△△	▲ 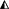 △	▲	△△△	△△△	▲	△	Unclear
Thillainadesan et al. [6]	▲△△	▲ 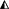 △	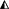 △△	▲△△	▲△△	▲△△	▲△△	▲ 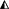 △	▲	△△△	△△△	△	△	Medium
Yan et al. [35]	▲ 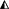 △	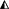 △△	–	▲▲▲	–	▲▲△	▲△△	▲▲▲	▲	△△△	△△△	△	▲	Unclear
Zhang et al. [36]	▲ 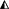 △	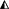 △△	–	▲▲▲	–	▲▲△	▲△△	▲▲△	▲	△△△	△△△	▲	▲	Low

△△△ = Score 0 out of 3

▲△△ = Score 1 out of 3

▲
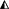
△ = Score 1.5 out of 3

▲▲▲ = Score 3 out of 3

## Discussion

### Principal findings

This systematic review evaluated the effectiveness of microlearning interventions in geriatric medicine education for healthcare professionals. The results presented suggest that microlearning is a promising and acceptable education modality, particularly suited for geriatric medicine teaching within fast-paced and dynamic clinical settings.

Despite the challenges of comparing heterogeneous interventions with different formats and implementation approaches, 90% (*n* = 36/40) of the educational outcomes were positive, and about one-third of these results (*n* = 12/40, 30%) were statistically significant. Moreover, all interventions that assessed learner attitudes (satisfaction, confidence) reported positive results, suggesting that microlearning could have an increasing role within the modern education framework. This is supported by existing literature that demonstrates an increasing preference for digital and interactive educational modalities [37] and that this is associated with increased intrinsic motivation [38, 39] and improved self-confidence, particularly with daily engagement [40]. The effectiveness of microlearning may be influenced by learner and contextual factors, including digital literacy, access to educational resources and individual learning preferences. These variables were not reported across included studies and may contribute to heterogeneity in outcomes, particularly at higher Kirkpatrick levels where evidence remains limited.

Of the seven studies [24, 26, 29–31, 33, 36] that specifically assessed Level 1 outcomes such as satisfaction, confidence and practicability, five of these studies investigated microlearning as a standalone intervention rather than part of a multimodal educational strategy. Gesin et al. [29] formulated a staged introduction of a didactic intervention after the initiation of a microlearning intervention, which demonstrated improvements in both attitudes and confidence at each stage of implementation. Existing literature into the ideal educational delivery approach remains mixed. One study demonstrated a preference for multimodal approaches in undergraduate medical students, but unimodal approaches in postgraduate students with a preference for strategic learning approaches that focus on time management [41]. Another study suggested that a preference for multimodal teaching increased with years of education [42]. Further larger-scale studies may better characterise the optimal approach, although it is likely a tailored approach to individual learners should be considered.

All the interventions that examined knowledge outcomes (i.e. Kirkpatrick Level 2 outcomes) demonstrated an improvement in short-term knowledge retention post-intervention; however, the practice effect commonly seen in before-and-after studies preclude a definitive causal relationship between the intervention and outcome of improved knowledge to be conclusively drawn [43]. Additionally, longer-term retention of knowledge outcomes post-intervention were not evaluated in any of the studies, and thus sustainability of the interventions in improving clinician knowledge and subsequently patient care cannot be ascertained with certainty. A previous study specifically assessing effects of gamification on knowledge retention [39] demonstrated increased activation of midbrain and ventral striatum regions on functional magnetic resonance imaging leading to improved intrinsic motivation, and this was subsequently associated with increased dopamine release, as well as limbic and hippocampal activity, which may promote longer-term memory recall.

There was a paucity of studies that evaluated improvements in Levels 3 and 4 Kirkpatrick outcomes (behaviour changes and patient health outcomes, respectively). Of the eight Level 3 outcomes assessed, only four focused on changes in behaviour related directly to patient care, namely prescribing rates of antipsychotics [24], analgesia [34], laxatives [34] and preoperative screening [6]. The remaining outcomes pertained to improvements in clinical documentation rather than direct care [24, 32, 36]. While meta-analysis indicated significant improvements in delirium knowledge and recognition across combined Kirkpatrick outcome levels, only one study assessed this at a Level 4 standard with direct patient implications [28]. This is a similar trend observed across other studies on microlearning within the healthcare setting [5, 37], with a scoping review by De Gagne et al. [5] focusing on healthcare students rather than clinicians showing over 80% of studies assessed Levels 1 and 2 outcomes, compared to only 29% and 0% assessing Levels 3 and 4 outcomes, respectively. This review has identified the gap where further research is needed that focuses on higher-level clinical and patient-centred outcomes. This is particularly relevant in the care of older adults, where multimorbidity and complexity make it difficult to isolate the effects of educational interventions, yet where meaningful clinical outcomes are most important.

### Limitations and future directions

Despite positive findings across most studies, extrapolating concrete conclusions about the existing effectiveness and acceptability of microlearning within this clinical context is impacted by the significant heterogeneity in study topics, target clinicians, intervention complexity and outcome measurement tools. This is further compounded by the relatively small number of publications available to date which precludes high-powered subgroup analysis to identify correlatory or causal effects. Additionally, consideration must be given to the possibility of the Hawthorne effect contributing to positive findings. The effectiveness of these interventions when implemented in routine clinical settings without dedicated educational champions remains uncertain.

Most studies focused on delirium and/or dementia outcomes, without any studies investigating other common geriatric syndromes such as falls, osteoporosis or frailty. Furthermore, there were no studies that had head-to-head comparison of microlearning against traditional didactic methodologies. In all studies that had a control group, the control was no formal educational intervention, in keeping with a common limitation of pre-post implementation studies. This limits conclusions that may be able to be drawn regarding the relative effectiveness of various teaching modalities. While microlearning supports discrete knowledge acquisition, care of older adults requires integration of multiple interacting conditions and development of clinical reasoning, and is therefore best viewed as complementary to, rather than a replacement for, multimodal clinical training.

There was also some variability in how validated tools for knowledge assessment were adapted to suit contemporaneous workplaces. For instance, multiple studies assessed delirium knowledge using a modified version of Hare et al.’s [44] original delirium knowledge questionnaire (DKQ) [26–28, 33]. While this may have risked invalidating the assessment tools, it likely provided a more helpful and practical snapshot of the impact of the intervention within the specific clinical context in which each study was undertaken. Modification of validated tools to improve relevance to a particular setting has also been incorporated in studies targeting medical student education rather than that of postgraduate clinicians [45].

As discussed previously, there was a marked preponderance towards Levels 1 and 2 outcomes, making up 65% of the included studies, and they were generally of moderate study quality. Most were small-scale studies, with only two interventions trialled at more than one site [31, 33], and both of which only assessed low-level Kirkpatrick outcomes. The transferability of interventions and their potential practical impact on direct patient outcomes requires further investigation with ideally multisite randomised controlled trials and interventions with staggered introduction to allow for better delineation of specific microlearning and/or didactic components that contribute best to learning outcomes. This is a barrier seen across multiple healthcare disciplines and is not restricted just to the use of microlearning within geriatric medicine [5].

The findings of this study were presented to the Patient and Public Involvement Panel at our research institution, comprising older adults and carers, including adults living with dementia. The Panel expressed the view that high-quality studies focusing on patient-centred outcomes (Kirkpatrick Level 4) would have greater practical value. Overall, there was generally positive feedback on the potential role for microlearning in clinical geriatric medicine. Beyond its time- and potential cost-effectiveness, the board commented on the adaptability and versatility of microlearning, particularly its ability to be implemented on an *ad hoc* basis to address specific clinical or practical knowledge gaps. For example, one panel member suggested a microlearning intervention on osteoporosis management targeted towards junior doctors attending to patients after a hip fracture. Inclusion of clinician learners in feedback discussions would have provided complementary insights to the utility of microlearning.

Despite growing interest in microlearning and optimistic findings from small-scale studies, there is little data regarding its cost-effectiveness. While microlearning is often considered time-efficient and potentially cost-saving, particularly when delivered digitally, its true economic value remains uncertain. The costs associated with development, implementation and maintenance of digital infrastructure require clearer quantification. This is an important consideration given the significant financial investments in healthcare education; for example, postgraduate medical and dental education alone accounted for approximately £1.8 billion in the UK in 2014–2015 [46].

## Conclusion

This review has highlighted that microlearning is emerging as a potentially effective and scalable tool for geriatric medicine education among healthcare professionals, particularly in fast-paced hospital settings. As healthcare becomes more technology-driven, microlearning could meet the need for accessible, on-demand learning. Large-scale, standardised and comparative studies are needed to assess knowledge retention, behavioural change and patient outcomes across a range of geriatric syndromes to determine whether this teaching modality improves clinical practice and outcomes for older patients, compared with other teaching and learning methods.

## Supplementary Material

aa-25-3654-File002_afag129

## Data Availability

Research data supporting the findings of this study are available from the corresponding author upon reasonable request.
