# Supplementary material for: Microlearning to teach geriatric principles in hospitals: a systematic review and meta-analysis
Source: Age Ageing. 2026 May 17;55(5):afag129. doi: 10.1093/ageing/afag129 (PMC13180271; doi:10.1093/ageing/afag129)
Supplement: aa-25-3654-File002_afag129 [file aa-25-3654-file002_afag129.docx]

**Microlearning to teach geriatric principles in hospitals: A systematic review and meta-analysis**

Appendix 1. MEDLINE search strategy

| Ovid MEDLINE(R) ALL <1946 to April 26, 2023>  1 (microlearn* or micro-learn* or microteach* or micro-teach* or microeducat* or micro-educat* or microunit* or micro-unit* or microskill* or micro-skill* or microlectur* or micro-lectur* or microcontent* or micro-content* or microformat* or micro-format* or just-in-time learn* or just-in-time train* or bite-size learn* or bite-size train*).mp. 926  2 (digital* or cell phone* or mobile phone* or smartphone* or smart phone* or iphone* or android* or mobile* or app or apps or mobile app* or text messag* or instant messag* or blog* or podcast* or webcast* or gamificat* or video gam* or video* or videoconferenc* or module* or social media* or online* or website* or electronic* or portable* or technolog* or computer-assisted instruct* or simulat*).mp. 2670380  3 exp cell phone/ or videoconferencing/ or social media/ or blogging/ or gamification/ or mobile applications/ or computer-assisted instruction/ or simulation training/ or electronic supplementary materials/ or digital technology/ 65770  4 2 or 3 2670380  5 (learn* or elearn* or e-learn* or teach* or educat* or course* or train* or curricul*).mp. 2867467  6 exp learning/ or teaching/ or education/ or education, professional/ 497368  7 5 or 6 3057126  8 4 and 7 398877  9 1 or 8 399544  10 (geriatric* or gerontolog* or ageing* or aging* or older* or elder* or senior* or frail* or falls* or dement* or deliri*).mp. 1371906  11 exp geriatrics/ or geriatric assessment/ or aged/ or aging/ 3569344  12 10 or 11 4177212  13 9 and 12 45310  14 (hospital* or acute care or ward).mp. 1951865  15 exp hospitals/ 315256  16 14 or 15 1969645  17 13 and 16 8263  18 (medical staff* or nursing staff* or nurs* or doctor* or nutritionist* or dietitian* or occupational therap* or pharmac* or physiotherap* or physical therap* or physician* or social work* or speech patholog* or health professional* or clinician*).mp. 6128341  19 (exp internship/ and residency/) or medical education, graduate/ or health personnel/ or medical staff/ or nursing staff/ or nutritionists/ or occupational therapists/ or personnel, hospital/ or pharmacists/ or physical therapists/ or physicians/ or social work/ 317353  20 18 or 19 6228876  21 17 and 20 3937  22 limit 21 to english language 3745 |
| --- |
